# Symmetry fundamentalism in quantum mechanics

**DOI:** 10.1007/s11098-021-01634-z

**Published:** 2021-04-02

**Authors:** David Schroeren

**Affiliations:** Department of Philosophy, University of Geneva, Rue de Candolle 2, 1211 Genève 4, Switzerland

**Keywords:** Symmetry, Fundamentality, Ontology, Quantum mechanics, State space, Particle physics

## Abstract

Modern particle physics suggests an intriguing vision of physical reality: we are to imagine the symmetries of the world as fundamental, whereas the material constituents of the world (such as particles and fields) are ontologically derivative of them. This paper develops a novel ontology for non-relativistic quantum mechanics which gives precise metaphysical content to this vision.

## Introduction

The goal of this paper is to develop a novel metaphysics for fundamental physics, a new way to think about what physics tells us about fundamental physical reality—one that challenges what is perhaps the default approach to theorizing about ontology. According to this default view, a physical ontology tells us about the fundamental material constituents of the world: roughly, things like particles or fields; entities that are contained in physical spacetime and whose behavior is determined by the laws of nature; objects from which everything else is built. This kind of view is reflected in a wide range of influential positions in metaphysics and philosophy of physics, such as Democritean atomism, Aristotle’s view of the world as a plenum, the Lewisian notion of a Humean mosaic and the concept of a local beable in the philosophy of physics.

But modern physics seems to suggest a very different vision of fundamental reality, one that differs quite drastically from this default approach: according to this vision, the world has no fundamental material constituents at all. This might seem fairly counterintuitive: at no point in the history of science has fundamental physics told us more about the material constituents of the world than it does today. To some, the suggestion that physics is not fundamentally about the world’s material constituents will come as something of a surprise.

However, this surprising vision and our astonishing achievements at describing the world’s material constituents have the same source. In the second half of the 20th century, fundamental physics experienced a major breakthrough in the way claims about the material constituents of the world could be derived. Physicists noticed that almost everything about particles, their essential properties and their dynamical behavior could be predicted just on the basis of claims about *symmetries* (such as Leibniz shifts). This breakthrough amounted to nothing less than revolution: *ask not what the world contains but what its symmetries are.*^1^

It is just this inferential success of symmetries that has been regarded as underwriting the vision that material objects aren’t fundamental. We are to imagine the most fundamental level of the physical world as involving symmetries, whereas the physical entities that we would ordinarily have thought of as the fundamental building blocks of the physical world—such as elementary particles or fields—are ontologically derivative of these aspects. Call this thesis *symmetry fundamentalism*. It has been echoed by influential physicists: for example, Steven Weinberg has argued that “symmetries are fundamental”^2^ and thus that “at the deepest level, all we find are symmetries and responses to symmetries,”^3^ whereas “matter [...] loses its central role in physics.”^4^ According to Werner Heisenberg, we should “replace the concept of a particle” with “the concept of a fundamental symmetry”, and “what we have to look for are not fundamental particles but fundamental symmetries.”^5^

The move from the inferential centrality of a notion to its metaphysical fundamentality is familiar. When claims about a notion are ubiquitous and powerful as an inferential tool, we are naturally tempted to consider whether this notion should be regarded as metaphysically fundamental. Doing so would be a powerful vindication of those inferential practices: on this line of thought, the notion of symmetry is inferentially useful precisely *because* it is metaphysically fundamental and so is involved in the bottom-line story of physical reality that fixes everything else.

Nonetheless, it is far from obvious what picture of fundamental reality is suggested by symmetry fundamentalism. Symmetries in physics are introduced as part of the mathematical formulation of physical theories. But symmetry fundamentalism is not the thesis that physical reality literally consists in mathematical objects; it is a thesis about the physical constituents of fundamental reality described in terms of those objects. To give precise content to symmetry fundamentalism, we therefore need to provide a physical ontology: a hypothesis about the physical objects, properties and relations that makes transparent which physical entities correspond to symmetries and that illuminates how things like particles and fields are ontologically derivative of these entities.

The goal of this paper is to do just that: I develop an ontology that gives symmetry fundamentalism a precise metaphysical underpinning. I do this in the context of non-relativistic quantum mechanics.^6^ The main reason for this choice is that this framework involves much fewer technical challenges than more recent theories of fundamental physics while exhibiting all the features on which the relevant symmetry-fundamentalist claims are based. This highlights a key advantage of the symmetry-fundamentalist approach to ontology. The relevant inferences can be found not only in virtually every successful quantum theory developed since the 1920s, but also in some of our best proposals for a quantum theory of gravity. This suggests that a precise statement of a symmetry-fundamentalist ontology for non-relativistic quantum mechanics can serve as a template for ontological theorizing about future physics.

I said that our task is not just to articulate a symmetry-fundamentalist ontology, but also to make good on the claim that things like particles or fields are ontologically derivative of the physical counterparts of symmetries. I will do this by demonstrating that every relevant proposition about the former can be defined by or expressed in terms of a proposition that is only about the latter. By the ‘relevant’ propositions I just mean those propositions that are expressible in the relevant materialistic theory, i.e. the theory according to which the material constituents of the world (such as particles or fields) are fundamental. For the purposes of this paper, I will assume that the relevant family of materialistic theories are those whose only physical posit is a fundamental field corresponding to the wave function, or *wavefunction monist* theories. The goal, then, is to show how every proposition about this fundamental field expressible in the formalism of wavefunction monist theories can be defined in terms of some fundamental proposition.

Note that this challenge is distinct from showing how claims about macro-level objects such as tables, chairs, and measurement devices can be derived from a fundamental ontology consistent with quantum mechanics. This is one of the guises of the notorious quantum measurement problem, and I will have nothing new to say about it in this paper. Since the symmetry-fundamentalist ontology I articulate reproduces the more familiar wavefunction monist ontology, a symmetry-fundamentalist account of macro-ontology can be parasitic on any workable wavefunction monist response to the quantum measurement problem.

I proceed as follows. Section 2 introdcues the notion of a *state-space symmetry* and explains why this notion of symmetry is the target of symmetry fundamentalism. In Sect. 3 I develop *ray substantivalism*, an ontology for non-relativistic quantum mechanics that vindicates symmetry fundamentalism. I then taxonomize possible versions of ray substantivalism in Sect. 4. Section 5 contrasts ray substantivalism with major existing views about the ontology of quantum mechanics and addresses some potential objections.

## State-space symmetries

The goal of this section is to introduce the notion of a state-space symmetry and to explain why it is this notion of symmetry that is operative in symmetry fundamentalism.

Abstractly, a symmetry of a mathematical space is a *structure-preserving automorphism* on that space: a transformation which preserves the structure of that space. For example: symmetries of Euclidean space are Euclidean isometries—automorphisms of Euclidean space that preserve the Euclidean metric. And symmetries of Minkowski space are automorphisms of Minkowski space that preserve the Minkowski metric.

The symmetries operative in symmetry fundamentalism are structure-preserving automorphisms of a different space altogether: the mathematical space that characterizes *state space*, the space of possible instantaneous states of the world. Automorphisms of this kind are also known as *state space symmetries*.^7^ In classical particle mechanics, state space is characterized in terms of a mathematical space known as *phase space*, and phase-space symmetries are *symplectomorphisms*: phase-space automorphisms that preserve the symplectic structure of phase space. In quantum mechanics, state space is characterized in terms of Hilbert space, and symmetries of this space are *unitary transformations*: Hilbert-space automorphisms that preserve the inner product.

State-space symmetry and dynamical symmetry are distinct notions: a dynamical symmetry is a state-space symmetry that preserves the laws. More precisely: a state-space symmetry *T* is a dynamical symmetry iff for every dynamically allowed trajectory *h*, there is another dynamically allowed trajectory $h'$ such that every point on *h* gets mapped by *T* to a unique point on $h'$. So, every dynamical symmetry is a state-space symmetry, but not vice versa.^8^

I should therefore explain why it is the notion of a state-space symmetry, rather than the notion of a dynamical symmetry, that is operative in symmetry fundamentalism. First of all, whether a given state-space symmetry is a dynamical symmetry plays no role in the relevant symmetry-based inferences; and if a dynamical symmetry figures in such an inference, it does so only in virtue of the fact that it is a state-space symmetry. Two examples: First, the only symmetry-related premise in the characterization of spin in non-relativistic quantum mechanics is that rotations are state-space symmetries.^9^ Similarly, the only symmetry-related premise in the classification of elementary particle kinds in terms of mass and spin is that relativistic boosts, rotations, and shifts (also known as *Poincaré transformations*) are state-space symmetries.^10^ Both are paradigmatic examples of symmetry-based inferences that motivate symmetry fundamentalism; but neither requires an assumption about whether the state-space symmetries in question also count as dynamical symmetries.^11^

Moreover, note that the notion of a state-space symmetry is also distinct from the notion of a *spatial* or *spacetime symmetry*: a symmetry of the physical space that contains the material constituents of the world, such as particles, fields, tables and chairs. We already encountered one kind of spatial symmetry: the symmetries of Euclidean space. Other spaces of this kind are Minkowski space or (more generally) Pseudo-Riemannian manifolds.

To be sure: there are special physical systems whose state-space, dynamical, and spacetime symmetries coincide. For example, consider a classical Newtonian particle world that contains exactly one particle in three-dimensional Euclidean space and suppose that its dynamics are governed by a Hamiltonian of the form $H= \frac{p^2}{2m} + V(x)$, where *V*(*x*) is a spherically symmetric potential. In this world, rotations are symmetries of all three kinds: rotations are spatial symmetries (since they preserve the Euclidean metric); they are state-space symmetries (since they preserve state-space structure); and they are dynamical symmetries (because they leave *H* invariant).

These kinds of assumptions are often tacit in standard presentations of mathematical connections on which the symmetry-based metaphysical theses are based.^12^ However, these assumptions do not hold in most cases of interest. For a classical *n*-particle world, one fairly generic kind of state-space symmetry is a rotation of an individual particle that holds fixed all other particles. But rotations of individual particles do not generally count as a dynamical symmetries: typical *n*-particle Hamiltonians are not invariant under such transformations. (Intuitively, this is confirmed by the fact that there are many ways of rotating an individual particle that result in empirically distinguishable situations.)

Similarly, spatial rotations of individual particles in an *n*-particle world cannot be subsumed under spatial symmetries of Euclidean space. A rotation of Euclidean space about a fixed point can be thought of as a map that assigns to each point of Euclidean space its image under that rotation; rotations are *permutations* of points in Euclidean space. Think of a rotation of Euclidean space containing *n* particles as a permutation of which locations are occupied by which of the *n* particles. Since rotations preserve the Euclidean metric, these permutations do not change particle distances. By contrast, a rotation of an individual particle changes the spatial location of that specific particle while holding the spatial locations of other particles fixed; so, rotations shift that particle both relative to the other particles *and* relative to the points of the underlying Euclidean space. *A fortiori*, rotations that are state-space symmetries are generally not equivalent to permutations of points of Euclidean space. Rotations as state-space symmetries are equivalent to rotations of Euclidean space only in the special case of rigid, uniform rotations of all *n* particles, or in the special case of single-particle worlds. To illustrate: in a world containing only a single classical particle in Euclidean three-space, changing the spatial location of that particle by a rotation about some axis (a state-space symmetry) is tantamount to a permutation of the points of Euclidean space (a spatial symmetry). This is not true in general.

The upshot is twofold: first, although state-space symmetries have systematic relationships with dynamical and spacetime symmetries in certain special cases, the notions are distinct. Second, even in the special cases where these three notions coincide, the fact that a given transformation figures in the relevant symmetry-based claims is due only to its status as a state-space symmetry.

Let me stress again that state-space symmetries are mathematical functions: structure-preserving automorphisms on the mathematical space that characterizes state space. To vindicate symmetry fundamentalism, we need to articulate an ontology that makes transparent what physical entities correspond to these functions. This is what I turn to now.

## Symmetry-fundamentalist ontology

In this section, I develop a symmetry-fundamentalist ontology for non-relativistic *N*-particle quantum mechanics. The mathematical framework I take as the starting point consists of two things: first, a Hilbert space used to characterize the quantum states of the world. Second, a range of Hermitian operators that encode the physical structure among quantum states; more on this in a moment. Call this formalism the *Hilbert space framework*.^13^ Some version of it is part of the formalism of virtually every existing theory of non-relativistic quantum mechanics, including Bohmian mechanics, Everettian quantum mechanics, and dynamical-collapse theories.

A Hilbert-space automorphism can be thought of as a relation among Hilbert space vectors: the relation that obtains between vectors *x* and *y* iff the automorphism maps *x* to *y*. A natural way to implement fundamentalism about state-space symmetries is therefore to posit fundamental relations that correspond to these automorphisms.

This suggests that the relata of those relations should be regarded as fundamental entities as well. On a straightforward understanding of fundamentality, an entity is fundamental *if and because* it figures in some fundamental proposition.^14^ Thus, if the physical counterpart of a state-space symmetry is a fundamental relation, then this is so because it occurs in some fundamental proposition. But the sorts of propositions we express in terms of state-space symmetries also mention the *relata* of those relations: whatever physical entities correspond to Hilbert space vectors. On this line of thought, the fundamentality of the relations corresponding to state-space symmetries implies the existence of a fundamental space whose points are the relata of those relations.

It is natural to think of this fundamental space as *state space*, the space of quantum states of the world. In the context of non-relativistic *N*-particle quantum mechanics, this raises an important choice point. It is standard to assume that quantum states correspond to Hilbert space rays rather than to Hilbert space vectors. On this view, quantum state space corresponds not to linear Hilbert space but to *projective* Hilbert space.

Notwithstanding this standard assumption, physicists and philosophers alike routinely appeal to linear Hilbert space structure in their theoretical inferences; indeed, reformulating the entire technical machinery of quantum mechanics in the language of projective Hilbert spaces is a challenging task in its own right.^15^ Articulating an ontology that is fundamentalist about projective Hilbert space therefore faces obstacles that go beyond the usual difficulties of metaphysical theorizing about some theory of mathematical physics: it needs to engage with (and perhaps even develop new aspects of) a mathematical machinery that is non-standard in both physics and philosophy. In light of these considerations, some may be moved to more seriously entertain a seemingly naïve ontology that breaks with orthodoxy and posits states that stand in one-to-one correspondence to Hilbert space vectors.

Although the project of developing a state-space fundamentalist ontology for projective Hilbert space is beyond the scope of this paper, the view I propose strikes a compromise between this ambitious project and the unorthodox view. According to the ontology I call *ray substantivalism*, there is a fundamental space that has the structure of linear Hilbert space. But physical states are identified not with points in this space but rather with certain mereological constructions out of these points—entities that are in one-to-one correspondence with rays. To develop this view, I will first give an explicit statement of the more simple-minded ontology, a view I call *vector substantivalism*. This will serve as the basis for the subsequent articulation of ray substantivalism.

It is worth stressing right at the start that vector and ray substantivalism are importantly different from proposals that go by the label of *wavefunction realism*. According to these proposals, the main role of Hilbert space is to characterize the physical fields on some underlying fundamental space, fields that are represented by the *wavefunction*. Within the Hilbert space framework, the wavefunction of an abstract Hilbert space ‘ket’ $|\varphi \rangle$ is defined as $\varphi (q_1,...,q_N) = \langle q_1,...,q_N|\varphi \rangle$, where $|q_1,...,q_N\rangle$ is a simultaneous eigenvector of the position operators and *N* is the number of particles.^16^ Vector and ray substantivalists take a different view of the role of Hilbert space: instead of being a vehicle for characterizing physical fields on some underlying fundamental space, Hilbert space corresponds to a fundamental space in its own right—the space whose points are in one-to-one correspondence with abstract Hilbert space vectors.

### Vector substantivalism

With those clarifications out of the way, we can move on to the first step: a precise statement of vector substantivalism. I will proceed in analogy to the symmetry-fundamentalist ontology for classical particle mechanics developed in (Schroeren 2020).

The classical account has two main elements: first, the specification of a fundamental space with the structure of a classical phase space; and second, the specification of a sparse collection of fundamental properties and relations such that every relevant materialistic proposition (i.e. every proposition about the positions and momenta of classical particles) can be defined in terms of those fundamental properties and relations, together with the structure of the fundamental space. The key elements in this collection are two dyadic relations that correspond to the classical state-space symmetries of shifting the positions and momenta of individual particles:

**Classical position-almost-sameness.** A dyadic relation. Relates two points iff they agree about the momenta of all particles and about the positions of all but one particle.

**Classical momentum-almost-sameness.** A dyadic relation. Relates two points iff they agree about the positions of all particles and about the momenta of all but one particle.

The scheme by which every materialistic proposition can be defined in terms of these primitives exploits the mathematical correspondence, within Hamiltonian mechanics, between position- and momentum phase-space coordinate functions of individual particles on the one hand and the generators of momentum- and position-shift phase-space symmetries on the other. The specifics of this scheme won’t matter in what follows;^17^ for present purposes, I merely want to point out that the central ontological primitives I’ll introduce in this paper are quite similar to classical position- and momentum-almost sameness. Moreover, the scheme by which the primitives of vector and ray substantivalism serve to define every relevant materialistic proposition exploits an analogous mathematical correspondence between the position- and momentum-variables of quantum systems (usually encoded in terms of position- and momentum-operators) on the one hand and unitary momentum- and position-shift symmetries on the other.

Just like its classical counterpart, vector substantivalism has two main elements: first, a fundamental space with the structure of state space; and second, a sparse collection of primitives such that every relevant materialistic proposition can be defined in terms of them, together with the structure of the fundamental space.

To begin with, the vector substantivalist posits a range of *Points*—first-order individuals that stand in one-to-one correspondence with Hilbert-space vectors—as well as instants of time. Points are conceived of as quantum states; that is, they are the sorts of entities that can be ‘actualized’ at various instants of time. This is captured by the following posit:

**Point actuality.** A dyadic relation. Relates a Point *x* and an instant of time *t* iff *x* is actualized at *t*.

For Points to behave like states, vector substantivalism includes the following basic law: necessarily, for every instant of time there is exactly one Point that is actualized at it.^18^^,^^19^

Over and above Points, times, and Point actuality, the vector substantivalist requires primitives that confer on Points the structure of a Hilbert space. A nominalistic (or *intrinsic*) account of this structure is beyond the scope of this paper.^20^ Here I will assume that, as far as state-space structure on Points is concerned, we can help ourselves to ontological primitives that take complex numbers as arguments. Together with standard Hilbert space axioms, the following primitives confer Hilbert space structure on states:

**1. Sum.** A triadic relation. Relates Points *x*, *y*, and *z* iff *x* is the sum of *y* and *z*. Write $x=y+z$.

**2. Multiplication.** A triadic relation. Relates Points *x* and *y* and a complex number *c* iff *x* is a multiple by *c* of *y*. Write $x=cy$.

**3. Inner Product.** A triadic relation. Relates Points *x*, *y* and a complex number *c* iff *c* is the inner product of *x* and *y*. Write $c=(x,y)$.

The main challenge for the vector substantivalist is to show that every relevant materialistic proposition can be defined by or expressed in terms of some fundamental proposition about Points. The fundamental properties and relations that capture Hilbert space structure among Points are insufficient for this task, since any given pattern in these primitives is compatible with mutually exclusive materialistic propositions. For example, a Hilbert space of appropriate dimensionality can be used both to describe a world that contains physical systems with spin and a world that contains no such systems.^21^ To define every relevant materialistic proposition in fundamental terms, the vector substantivalist therefore requires further ontological primitives. This is the second main element of vector substantivalism.

I will introduce these primitives in terms of their mathematical characterizations; but it is important to emphasize that these characterizations are not metaphysical definitions. It should go without saying that ontological primitives cannot be defined in more fundamental terms, let alone in terms of their mathematical description. As is common in metaphysical theorizing, novel fundamental posits must be justified by their role in an account that highlights their explanatory and inferential connections to more familiar notions. This is precisely my goal here: it will shortly become clear that our primitives figure in an account according to which every relevant proposition about the physical counterparts of wavefunctions is definable by some proposition about these primitives.

To begin with, let me revisit a remark I made earlier: that Hermitian operators on Hilbert space are used to encode the physical structure on quantum states. This idea is most transparent from the wavefunction realist perspective. We already saw how the wavefunction is defined in terms of the eigenvectors of the position operators: the wavefunction of any ket $|\varphi \rangle$ is $\varphi (q_1,...,q_N) = \langle q_1,...,q_N|\varphi \rangle$, where $|q_1,...,q_N\rangle$ is a simultaneous eigenvector of the position operators. This procedure doesn’t just define some particular wavefunction; it defines *all possible* wavefunctions.^22^ In other words, it yields a range of wavefunctions that characterize the possible instantaneous arrangements of the physical field over the fundamental space, arrangements that possibly differ in the property that the field assigns to a given location in this space. In this sense, Hermitian operators designated as ‘position operators’ encode the physical structure of the fundamental field posited by the wavefunction realist.

In the vector substantivalist setting, the thought is quite similar. Every Point can be mathematically characterized in terms of a linear combination of eigenvectors of the relevant Hermitian operators. This can be illustrated at the level of mathematical description: a ket $|\varphi \rangle$ can be specified in terms of position eigenvectors as

1 $$\begin{aligned} |\varphi \rangle = \int _{-\infty }^{\infty }|q_1,...,q_N\rangle \langle q_1,...,q_N|\varphi \rangle dq_1...dq_N. \end{aligned}$$

Every difference between distinct kets therefore corresponds to a difference in some complex coefficients that figure in the expansions of those kets in terms of the simultaneous position eigenvectors. This illustrates how Hermitian operators encode *physical relations* among distinct Points: respects in which Points possibly differ from each other.

According to vector substantivalism, these physical relations correspond to state-space symmetries. To substantiate this thesis, we exploit a mathematical duality, known as Stone’s theorem, between state-space symmetries and Hermitian operators on Hilbert space.^23^^,^^24^ For ease of exposition, let me introduce some terminology. Say that each of the *N* real numbers that figure in simultaneous position eigenvectors such as $|q_1,...,q_N\rangle$ are values of the *position variables*. Correspondingly, for any $|q_1,q_2,...,q_N\rangle$ and $|q'_1,q_2,...,q_N\rangle$ such that $q_1\ne q_1'$, I will say that the two vectors *differ in the first position variable*. This extends to arbitrary linear combinations of eigenvectors. For example, the following vectors differ only in the first position variable:

2 $$\begin{aligned} |\varphi \rangle&= \int _{-\infty }^{\infty }|q_1,...,q_N\rangle \langle q_1,...,q_N|\varphi \rangle dq_1...dq_N \end{aligned}$$

3 $$\begin{aligned} |\phi \rangle&= \int _{-\infty }^{\infty }|q_1+a,...,q_N\rangle \langle q_1,...,q_N|\varphi \rangle dq_1...dq_N. \end{aligned}$$

Stone’s theorem says that there is a one-to-one correspondence between one-parameter unitary transformations and Hermitian operators. The mathematical details do not matter for our purposes; what’s important is that, if ${\hat{p}}$ is a momentum operator then

4 $$\begin{aligned} U(a) = e^{ia{\hat{p}}} \end{aligned}$$

is the unitary operator that implements a shift by amount *a* in the corresponding *position* variable. Similarly, if ${\hat{q}}$ is a position operator then

5 $$\begin{aligned} V(k) = e^{ik{\hat{q}}} \end{aligned}$$

is the unitary operator that implements a shift by amount *k* in the corresponding *momentum* variable. For example, the unitary operator $U_1(a)$ that acts on the simultaneous position eigenvector $|q_1,q_2,...,q_N\rangle$ as $U_1(a)|q_1,q_2,...,q_N\rangle = |q_1+a,q_2,...,q_N\rangle$ implements a shift in the first position variable. The action of shift operators on eigenvectors determines their action on arbitrary linear combinations of eigenvectors. For example, it is immediate from equations (2) and (3) that

6 $$\begin{aligned} |\phi \rangle = U_1(a)|\varphi \rangle . \end{aligned}$$

This suggests two relational primitives for vector substantivalism that are quite similar to the primitives of classical symmetry fundamentalism reviewed earlier. The idea is that we posit one fundamental relation that obtains between two Points when they are related by a shift in some position variable and one fundamental relation that obtains between two Points when they are related by a shift in some momentum variable:

**4. Position-almost-sameness.** A dyadic relation. Relates two Points just in case they agree about all position variables except for a shift in one position variable.

**5. Momentum-almost-sameness.** A dyadic relation. Relates two Points just in case they agree about all momentum variables except for a shift in one momentum variable.

I will now show that, for systems without spin, these two relations (together with the relations that confer Hilbert space structure on Points) are sufficient for specifying a range of fundamental propositions such that, for every proposition about the ontological counterparts of wavefunctions, one of these fundamental propositions defines it.

My strategy takes inspiration from the above-mentioned mathematical definition of wavefunctions in terms of kets: $\phi (q_1,...,q_N)= \langle q_1,...,q_N|\phi \rangle$. The main idea is that, for any fixed ket, this expression can be thought of as capturing a relation between simultaneous eigenvectors and the complex numbers. At the ontological level, the corresponding idea is that the physical counterpart of the wavefunction is some specific pattern in the inner product relation. To implement this idea, we need to define two pieces of derivative ontology: first, a property instantiated by a Point just in case its mathematical representative is a simultaneous eigenvector; second, physical entities whose surrogates are points of configuration space and points of momentum space, respectively.

I start with the ontological counterpart of a simultaneous eigenvector. Let a *position-almost-same subspace* be a space of Points such that any two Points in it are position-almost-same; and let a *momentum-almost-same-subspace* be a space such that any two Points in it are momentum-almost-same. Points in a given position- or momentum-almost-same subspace differ about the same position or momentum variables. Let *y*, *z* be Points that are position-almost same to some Point *x*, and suppose that the differences between *x*, *y* and the differences between *x*, *z* concern different position variables. It follows that *y*, *z* differ about more than one position variable and so at most one of *y* and *z* can be in the same position-almost-same subspace as *x*. (Similarly for momentum-almost-same subspaces.)

Next, we observe that, if $|p\rangle$ is an eigenvector of a momentum operator ${\hat{p}}$ then it is also an eigenvector of the unitary operator *U*(*a*) that implements shifts in the corresponding position variable:

7 $$\begin{aligned} U(a)|p\rangle = e^{iap}|p\rangle \end{aligned}$$

A similar fact holds for position eigenvectors: if $|q\rangle$ is an eigenvector of a position operator ${\hat{q}}$ then it is also an eigenvector of the unitary operator *V*(*k*) that implements shifts in the corresponding momentum variable:

8 $$\begin{aligned} V(k)|q\rangle = e^{ikq}|q\rangle \end{aligned}$$

In other words: kets that are eigenvectors of some *momentum* operator are such that the corresponding *spatial* shift operator maps them to complex scalar multiples of themselves; and momentum eigenvectors are the only kets with this property. Similarly, kets that are eigenvectors of some *position* operator are such that the corresponding *momentum* shift operator maps them to complex scalar multiples of themselves; and position eigenvectors are the only kets with this property. We can use this fact to define the following physical properties that correspond to the notion of an eigenvector:

**A. Position-eigenpoint.** A monadic property. Instantiated by a Point *x* iff for some momentum-almost-same subspace containing *x*, every Point in that subspace is a multiple of *x*.

**B. Momentum-eigenpoint.** A monadic property. Instantiated by a Point *x* iff for some position-almost-same subspace containing *x*, every Point in that subspace is a multiple of *x*.

The properties of being a position- or momentum-eigenpoint are instantiated by Points whose mathematical surrogates are eigenvectors of at least one position or momentum operator: for example,

9 $$\begin{aligned} |q_1,\varphi _2,...,\varphi _N\rangle = \int _{-\infty }^{\infty }|q_1,q_2,...,q_N\rangle \varphi _2(q_2)...\varphi _N(q_N)dq_2...dq_N \end{aligned}$$

is an eigenvector in the first position variable but not necessarily an eigenvector in other position variables. But to define the intrinsic counterparts of position and momentum wavefunctions, we also need properties instantiated by Points when their mathematical surrogates are *simultaneous* eigenvectors of all position or all momentum operators. These can be defined as follows:

**C. Simultaneous position-eigenpoint.** A monadic property. Instantiated by a Point *x* iff every Point momentum-almost-same to *x* is a multiple of *x*.

**D. Simultaneous momentum-eigenpoint.** A monadic property. Instantiated by a Point *x* iff every Point position-almost-same to *x* is a multiple of *x*.

The second task is to define entities whose mathematical surrogates are points of configuration space and points of momentum space, respectively. It may be tempting to think that simultaneous position- and momentum-eigenpoints are good candidates for this role. This temptation should be resisted: there are too many simultaneous position and momentum eigenpoints. This is most transparent at the level of mathematical description. Given some fixed coordinate system on configuration space, there is a one-to-one correspondence between points of configuration space and the eigenvalues of the simultaneous position-eigenvectors of position operators along the axes of this coordinate system. But there are many more distinct simultaneous position eigenvectors than there are distinct position eigenvalues: for any complex number *c*, $|q_1,q_2,...,q_N\rangle$ and $c|q_1,q_2,...,q_N\rangle$ are not just both simultaneous position eigenvectors; they are eigenvectors with identical position eigenvalues. This problem can be sidestepped using tools from mereology:

**E. Configuration space point.** A monadic property. Instantiated by *q* iff *q* is a fusion of Points and every atomic part of *q* is a simultaneous position eigenpoint and any two atomic parts of *q* are related by a complex scalar multiple.

**F. Momentum space point.** A monadic property. Instantiated by *p* iff *p* is a fusion of Points and every atomic part of *p* is a simultaneous momentum eigenpoint and any two atomic parts of *p* are related by a complex scalar multiple.

This gives the right result: for every position/momentum eigenvalue, there is a unique configuration/momentum space point: the point such that any two of its atomic parts have this eigenvalue.

We are almost ready to define the ontological counterparts of wavefunctions. The last observation we need to make is that wavefunctions are defined in terms of *normalized* simultaneous eigenvectors, rather than in terms of their complex scalar multiples. For example, we define the wavefunction of $|\phi \rangle$ in terms of vectors like $|q_1,q_2,...,q_N\rangle$ rather than in terms of $c|q_1,q_2,...,q_N\rangle$ where $c\ne 1$.

It is important that we take note of this fact. Recall: our strategy is to identify wavefunctions with certain specific patterns in the inner product relation. But whereas wavefunctions assign a complex number to every point of configuration or momentum space, the inner product takes Points as arguments rather than the fusions of Points. This means that, for any given Point *x* and configuration space point *q*, the atomic parts of *q* differ in the complex number assigned to them by the inner product. This raises the question as to which complex number the putative ontological counterpart of a wavefunction should assign to *q*. The answer is suggested by the mathematical observation in the previous paragraph: it is the number that bears the inner product relation to every *normalized* atomic part of *q*.

More precisely, let a Point *x* be *normalized* iff there is some complex number *c* of unit modulus such that $c=(x,x)$. The ontological counterparts of wavefunctions are then defined as follows:

**G. Configuration-space field of x.** A dyadic relation. Relates a complex number *c* and a configuration space point *q* just in case, for every normalized atomic part *y* of *q*, $c=(y,x)$.

**H. Momentum-space field of x.** A dyadic relation. Relates a complex number *c* and a momentum space point *p* just in case, for every normalized atomic part *y* of *p*, $c=(y,x)$.

According to vector substantivalism, the non-fundamental proposition we express when we specify the wavefunction of some Point *x* is the proposition that details the pattern in the configuration-space field of *x*: the proposition that specifies, for every configuration space point *q*, the complex number that is the value of the configuration-space field of *x* at *q*. (Similarly for momentum.) Since the configuration-space field of *x* is defined in terms of the primitives (1-5), it follows that every non-fundamental proposition about the ontological counterparts of wavefunctions statable in the Hilbert space framework can be defined by (or expressed in terms of) some fundamental proposition about relations whose mathematical surrogates are state-space symmetries. Vector substantivalism is therefore an instance of symmetry fundamentalism.

### Ray substantivalism

Vector substantivalism entails that Points are quantum states and is therefore at odds with the orthodox view that quantum states correspond to Hilbert space rays rather than to Hilbert space vectors. In this section, I sketch *ray substantivalism*, an ontology that respects this consensus. This view is not an attempt at the more ambitious project of fundamentalism about projective Hilbert space. Instead, it is the view that quantum states are identified with certain mereological fusions of Points.

Ray substantivalism is a fairly straightforward modification of vector substantivalism. The fundamental first-order posits are identical: they consist in Points and instants of time. Moreover, the ray substantivalist follows the vector substantivalist in positing Hilbert space structure (1–3) among Points as well as the relations of position- and momentum-almost-sameness (4,5). The central difference between the two views is the identification of the sorts of entities that can be actualized at various instants of time; that is, the entities that are identified with quantum states. Let a fusion *s* of Points be a *State* iff for any two atomic parts *x*, *y* of *s*, *x* is a multiple of *y*. According to the ray substantivalist, actuality is a fundamental relation between instants of time and States:

**State actuality.** A dyadic relation. Relates a State *s* and an instant of time *t* iff *s* is actualized at *t*.

In addition, the ray-substantivalist version of the basic law governing the actuality relation says that necessarily, for every instant of time there is exactly one State that is actualized at it.

The only other feature of vector substantivalism that the ray substantivalist needs to modify is the definition of configuration- and momentum-space fields. Definitions G and H associate a field with every Point. But for the ray substantivalist, this is too fine-grained: the physical entity that corresponds to wavefunctions should be such that there is one such entity for every State rather than one for every Point. The idea behind the following modified definitions is that the ray-substantivalist field of a State *s* is the vector-substantivalist field of the normalized atomic part of *s*.

**G′. Normalized configuration-space field of s.** A dyadic relation. Relates a complex number *c* and a point of configuration space *q* just in case *c* is assigned to *q* by the configuration-space field of the normalized atomic part of *s*.

**H′. Normalized momentum-space field of s.** A dyadic relation. Relates a complex number *c* and a point of momentum space *p* just in case *c* is assigned to *p* by the momentum-space field of the normalized atomic part of *s*.

This completes the outline of ray substantivalism. According to this view, at every instant of time, the propositions about the normalized configuration- and momentum-space fields statable in the Hilbert space framework are entailed by which State is actual at that instant, together with the proposition that details the pattern of properties of and relations among the atomic parts of the actualized State and other Points. Ray substantivalism agrees with vector substantivalism about the fundamental first-order posits, as well as about the fundamental structure on Points. But the two views disagree about the nature of quantum states as well as about the physical counterparts of wavefunctions.

Like vector substantivalism, ray substantivalism is an instance of symmetry fundamentalism: almost-sameness relations are fundamental relations that correspond to state-space symmetries; and every relevant proposition about the physical counterparts of wavefunctions is defined by some fundamental proposition about these relations.

Some readers might object that ray substantivalism does not seem like a genuine advance over vector substantivalism. Recall that the central motivation for the orthodox view (according to which quantum states correspond to rays rather than to vectors) is to avoid a problematic underdetermination implied by the competing view: since no experiment can distinguish between states of a quantum system that differ only by a global phase, the thesis that quantum states correspond to Hilbert space vectors implies that it is in-principle empirically inaccessible which quantum state is actual. However, it seems as if ray substantivalism achieves consistency with the standard view only at the cost of reproducing the underdetermination at the level of the atomic parts of States. On ray substantivalism, the actualized State contains a rich structure among its parts, a structure which seems in-principle empirically inaccessible. But this worry is misplaced. According to ray substantivalism, there is no in-principle experimental underdetermination of which State the world is in at any given instant of time. And given that the State of the world at some instant of time is known, the structure among its atomic parts can be inferred simply from the linear Hilbert space structure among Points. Ray substantivalism does not feature the sort of underdetermination that spells trouble for vector substantivalism.

## A taxonomy of ray-substantivalist views

The ontology I just outlined is not the only version of ray substantivalism. The goal of this section is to provide a taxonomy of the available options.

The range of possible ray-substantivalist views mirrors the varieties of wavefunction realism. There are two important respects in which the latter differ. The first concerns the fundamental space that is the container of the physical field characterized by the wavefunction. *Configuration-space realists* say that the fundamental field lives on configuration space, the 3*N*-dimensional space whose points we can intuitively think of as each fixing a distribution of *N* classical point particles in Euclidean three-space. *Multi-field realists*, by contrast, say that the fundamental field assigns a property to every *N*-collection of points of Euclidean three-space.^25^ The second line of disagreement is about whether fundamental reality is exhaustively described by the wavefunction. *Wavefunction monists* say ‘yes’. The most influential theories that answer this question in the negative are what I’ll call *local beable* theories. According to them, fundamental reality additionally consists in local beables: roughly, physical objects that are confined to a bounded region of the fundamental space.^26^

For example, GRW$_0$ is a configuration-space-realist version of wavefunction monism according to which the dynamical evolution of the fundamental field is given by a collapse equation.^27^ Bohmian mechanics is a local beable theory that has both a configuration-space-realist as well as a multi-field-realist variant. According to the former, there is a single ‘world-particle’ in configuration space. According to the latter, there are point-particles in three-space.^28^ The two theories agree that the evolution of the particulate content of the world is determined by the fundamental field which itself evolves according to the Schrödinger equation.^29^ GRW$_f$ and GRW$_m$, understood as local beable theories, have a configuration-space-realist and a multi-field-realist variant. Both variants posit physical entities (flashes or mass densities) distributed over the fundamental arena whose temporal evolution is determined by the fundamental field. Finally, there are two wavefunction monist options for Everettian quantum mechanics (EQM): a configuration-space-realist one and a multi-field-realist one.^30^

This taxonomy can be transposed more or less directly to ray substantivalism. The view I articulated in the previous section can be regarded as *configuration-space-realist monistic* ray substantivalism. It is monistic in the following sense: at every instant of time, physical reality is determined entirely by which State is actualized at that time, together with relations between the atomic parts of the actualized State and other Points. Moreover, the view is configuration-space realist: for every State, the ontologically derivative entity that corresponds to the wavefunction is a field on configuration space. Another possible monistic view is *multi-field-realist* ray substantivalism, in which the main ontologically derivative entity is a multi-field on physical three-space. Both versions of monistic ray substantivalism can be combined with dynamical laws of either the dynamical-collapse or the Schrödinger sort, resulting in variants of GRW$_0$ and EQM, respectively.

There are also ways to supplement monistic ray substantivalism with local beables. Here, I will focus on two Bohmian versions of this approach. The first version is what you might call *configuration-space-realist Bohmian ray substantivalism*: the view which (over and above the ray substantivalist primitives) posits a fundamental configuration space as well as a single Bohmian ‘world-particle’ located in it. An alternative is *multi-field-realist Bohmian ray substantivalism*, a view which includes the primitives of monistic ray substantivalism and additionally posits a fundamental physical three-space as well as a collection of point particles located in it. On both views, the dynamical evolution of the fundamental particle(s) are determined by the actualized State according to the guidance equation; and the dynamical law governing States is given by the Schrödinger equation. These views differ from monistic ray substantivalism in both their fundamental and their derivative ontology: they posit fundamental particles as well as points of the respective fundamental spaces and therefore have no need to lay down definitions of these fundamental spaces in terms of Points.

Although Bohmian versions of ray substantivalism are coherent candidate ontologies, there are three reasons to be skeptical. First, they have costs that they don’t share with their wavefunction-monist competitors. The Bohmians we’re imagining posit two fundamental spaces: the space of Points, plus whatever space contains the local beables. Wavefunction-realist Bohmians, by contrast, get by with positing just one fundamental space: the arena which contains both the Bohmian particles and the fundamental field. Both kinds of Bohmian ray substantivalism therefore seem less ontologically parsimonious than their wavefunction-realist counterparts.

Second, neither version of Bohmian ray substantivalism is an instance of symmetry fundamentalism. To be sure: these views posit almost-sameness relations among Points and are therefore consistent with the first conjunct of symmetry fundamentalism. But they are inconsistent with the second conjunct. The Bohmian particles are supposed to be ontologically primitive, metaphysically fundamental, not ontologically derivative of anything else. So, it is not the case that every relevant proposition about Bohmian particles can be defined in terms of some proposition about almost-sameness relations.

Third, there is a systematic relation between the symmetries of the fundamental space that contains the Bohmian particle(s) on the one hand and the relevant state-space symmetries on the other. For example, it is routine to define unitary actions of the rotation group *SO*(3) on Hilbert space in terms of an action of this group as Euclidean isometries.^31^ To be sure: the existence of this kind of mathematical definition does not force the Bohmian ray substantivalist to lay down a corresponding metaphysical definition of the relevant sameness relations on state space. But it does imply that these views face the additional burden of explaining how the almost-sameness relations on the space of Points hang together with the symmetries of the fundamental container of the local beables.

## Discussion

This section is devoted to contrasting ray substantivalism with three of the most influential approaches to the ontology of quantum mechanics: wavefunction realism in both its monistic and local beable varieties, as well as spacetime state realism. I also respond to some objections that may be leveled against views along the lines of ray substantivalism.

First, it is important to note that none of the three approaches is consistent with symmetry fundamentalism. Recall that symmetry fundamentalism is the conjunction of two claims: first, that state-space symmetries correspond to fundamental aspects of physical reality; and second, that the kinds of things we would have ordinarily regarded as the fundamental building blocks of the world, such as particles or fields, are ontologically derative from these fundamental aspects.

Existing proposals are inconsistent with the first conjunct: they do not posit fundamental entities that correspond to state-space symmetries. The most straightforward case is wavefunction realism in both monistic and local beable form. Theories of this type posit a fundamental physical field on the relevant fundamental arena, plus (perhaps) the distribution of local beables over this arena. On these views, nothing in the ontology corresponds to state-space symmetries.

Another proposal for the ontology of quantum theory is known as spacetime-state realism, one that differs quite significantly from wavefunction realism. We are to imagine fundamental physical reality as involving a distribution of physical properties over regions of spacetime: the *quantum states* of those regions.^32^ Mathematically, each region *i* is assigned a Hilbert space ${\mathcal {H}}_i$ and the Hilbert space ${\mathcal {H}}$ of the world is given by the tensor product of these Hilbert spaces: ${\mathcal {H}}= \bigotimes _i {\mathcal {H}}_i$. If the quantum state of the universe at time *t* is given by the density operator $\rho$ on ${\mathcal {H}}$, then the quantum state of spacetime region *i* at *t* is given by the partial trace $\rho _i = Tr_{i\ne j}\rho$.

There are at least two ways to understand this view. The first is more serious about the idea that quantum states are represented by operators on Hilbert spaces. On this line of thought, for every spacetime region *i* we posit a fundamental space corresponding to ${\mathcal {H}}_i$ as well as a fundamental relation on this space corresponding to $\rho _i$. This version of spacetime-state realism collapses into a form of ray substantivalism: an ontology according to which there is a collection of spaces of Points, together with certain privileged relations among those Points.

The similarity between ray substantivalism and this version of spacetime-state realism becomes even more transparent once we take notice of an observation made by Ismael and Schaffer (2020). Understood as fundamental entities, the quantum states assigned to spacetime regions smaller than the entirety of spacetime are ontologically redundant: as the above derivation of $\rho _i$ from $\rho$ suggests, the quantum state of any spacetime region is completely fixed by the state of the world as a whole, but not vice versa. According to what Ismael and Schaffer refer to as ‘spacetime-state realism streamlined’, the fundamental ontology includes only the quantum states of the world as a whole: exactly what the ray substantivalist refers to as States.

So, ray substantivalism and this version of spacetime-state realism are very similar indeed. The main difference concerns the nature of the physical structure on state space: according to ray substantivalism, this structure consists in almost-sameness relations; according to ‘spacetime-state realism streamlined’, it consists in the physical properties and relations on state space that correspond to certain Hermitian operators on Hilbert space.^33^ Hermitian operators, however, are not themselves state-space symmetries but *generators* of state-space symmetries. So this version of spacetime-state realism is not an instance of symmetry fundamentalism.

This is not the version of spacetime-state realism intended by its main proponents.^34^ According to the official version of the view, the fundamental properties assigned to spacetime regions correspond to abstract operator algebras. Their technical definition is not important here; what matters is that the operators in these algebras need not be construed as operators acting on a Hilbert space.^35^ While this version of spacetime-state realism is therefore not at risk of collapsing into ray substantivalism, the absence of Hilbert spaces from this formulation makes it hard to see how the operators that encode the relevant physical properties can be thought of as ‘acting’ on anything, much less as being state-space symmetries.

To be sure: for every Hilbert-space automorphism that counts as a state-space symmetry within the Hilbert-space framework, there is an automorphism of the relevant operator algebra within the spacetime-state realist formalism, and vice versa. However, once the relevant operator algebras have been specified, their symmetries are physically redundant, in the sense that the relevant physical structure is already exhaustively specified in terms of the operator algebra alone. Let me illustrate this point by analogy to a fact about the Hilbert-space framework we encountered earlier. Recall that, in this framework, the relevant physical structure on Hilbert space can be specified either in terms of self-adjoint operators or in terms of the unitary transformations they generate. But once we have made the choice (for example) to characterize the physical structure in terms of self-adjoint operators, Stone’s theorem implies that nothing is added by also specifying the corresponding unitary transformations: all the physical structure is already fixed by the self-adjoint operators. The situation with regard to the operator-algebraic variant of spacetime-state realism is similar: once the relevant operator algebras have been specified, there is simply no job to be done by the symmetries of the algebra. Spacetime-state realism therefore cannot properly be understood as an instance of symmetry fundamentalism.

The result is that none of the major extant proposals for the ontology of quantum theory are instances of symmetry fundamentalism.^36^ Insofar as compatibility with symmetry fundamentalism is concerned, monistic ray substantivalism therefore has some claim to be the only game in town. Those in the market for a view that provides a precise ontological basis for symmetry fundamentalism should seriously entertain monistic ray substantivalism as a candidate ontology for non-relativistic quantum mechanics.

Second, there are aspects of monistic ray substantivalism that might make it less attractive than its competitors. A minimal adequacy condition for an ontology for quantum theory is that it be compatible with what is sometimes referred to as the ‘manifest image’: the familiar world of medium-sized dry goods, such as tables, chairs, and measurement devices. If an ontology does not satisfy this constraint, it is plainly inconsistent with the empirical evidence. However, some philosophers have suggested that we should impose more demanding constraints on ontological theorizing. Any candidate ontology must not just *be consistent with* the manifest image, it must also be *reflective* of certain important aspects of this image. According to local beable approach to physical ontology, for example, the relevant feature to be reflected in a fundamental ontology is that macroscopic objects are local beables. For a hypothesis to be admissible and intelligible as a fundamental ontology, it must therefore posit local beables as fundamental entities.^37^

Those who subscribe to constraints of this sort are unlikely to be attracted to monistic ray substantivalism. It’s not just that the fundamental ontology does not include local beables. Since there is no fundamental physical spacetime, the notion of a spatiotemporally localized entity is not even expressible in fundamental terms.

However, this is a predicament that should be familiar from discussions of configuration-space-realist wavefunction monism.^38^ Both views posit a fundamental space that is nothing like the space of our ordinary three-dimensional experience: ray substantivalism posits a space of Points isomorphic to Hilbert space and wavefunction monism posits fundamental configuration space. Moreover, neither view posits fundamental local beables. The challenges facing monistic ray substantivalism and configuration-space wavefunction monism are thus rather similar: it must be shown how the manifest image can be recovered from a fundamental picture that is quite unlike it.

The question of whether a fundamental ontology for non-relativistic quantum mechanics is capable of recovering the manifest image is part of a broader family of issues known as the ‘quantum measurement problem’: roughly, the challenge of providing a picture of fundamental physical reality according to quantum theory that is consistent with and explains our total macroscopic evidence, including the behavior of measurement devices.^39^ As we saw earlier, monistic ray substantivalism is capable of expressing all relevant propositions about the physical field that the wavefunction monist regards as fundamental. This suggests that if wavefunction monism succeeds in solving the quantum measurement, then so does monistic ray substantivalism.^40^

Third, I’d like to address a few objections that might be levelled against the kind of ontology articulated in this paper. Although the view that Hilbert space corresponds to a fundamental space in its own right has occasionally been mentioned in the literature, an ontology that gives precise content to this view has not yet been articulated. One reason might be that views of this sort have been regarded as “unmotivated”.^41^ As I hope to have shown in this paper, this is untrue: ray substantivalism is the natural result of ontological theorizing that is metaphysically serious about the recent paradigm shift towards symmetry techniques in physics. To be sure: ray substantivalism departs significantly from more familiar ways of thinking about the ontology of quantum mechanics. This might explain why views along the lines of ray substantivalism have been dismissed as “bizzare” in the literature.^42^ But it should come as no surprise that metaphysical theorizing about fundamental physics might fail to validate the pre-theoretical intuitions that are informed by our exposure to the macroscopic realm—especially when it comes to a notion so far removed from our everyday experience as that of a fundamental state-space symmetry.

A more specific worry, due to Jill North, is that a fundamental space corresponding to abstract Hilbert space does not have enough structure from which “to recover the ordinary world of our experience.”^43^ The thought is that “Hilbert space does not support an objective, structural distinction between positions and other physical properties, like spin, in the way that the wavefunction’s space does.”^44^

This charge is ineffective against ray substantivalism: after all, the view builds in the requisite structural distinction between positions and momenta at the level of the ontological primitives. To be sure: there’s something right about North’s observation. Ray substantivalism privileges neither the normalized configuration-space nor the normalized momentum-space fields in the way that wavefunction realism does; they are ontologically on a par. But this does not imply that ray substantivalism does not have the resources to recover the manifest image—or at least, it does not follow that ray substantivalism fares worse in this regard than (for example) configuration-space wavefunction monism. In particular, it would be incorrect to say that ray substantivalism has *too little* structure: as we have seen, every relevant proposition that is fundamental according to the wavefunction monist can be defined in ray-substantivalist terms. If anything, it may appear as if ray substantivalism has *surplus* structure: in addition to the propositions about the configuration-space field taken as fundamental by the wavefunction monist, every proposition about momentum-space fields statable in the Hilbert space framework can also be defined in ray-substantivalist terms. But this appearance is misleading. As we saw earlier, the ray-substantivalist definition of the configuration-space field of some State essentially involves the notion of momentum-almost-sameness: namely, via the definition of a simultaneous position-eigenpoint. Momentum structure is not ontologically redundant within ray substantivalism.

Fourth, let me address the seemingly modal nature of vector and ray substantivalism. In physics, instantaneous states are introduced as exhaustive and maximally specific descriptions of the fundamental properties of the material constituents of the world at an instant. A physical state can therefore be thought of as representing an instantaneous way the world *could* be and therefore seems quite similar to a possible world.^45^ Since the space of Points has the structure of a state space—Hilbert space—this might seem to imply that vector and ray substantivalism are committed to a form of modal realism.

This impression is incorrect. According to vector or ray substantivalism, states aren’t maximally specific and exclusive descriptions of anything: they are fundamental, ontologically primitive entities. Whatever modal intuitions we might have had about states *qua* exhaustive and maximally specific descriptions, they no longer apply once we regard states as fundamental entities. Both vector and ray substantivalism conceive of the space of Points as a fundamental space, as the fundamental arena in which the history of the world unfolds. If Points (or relevant mereological fusions of them) are deserving of a characterization in modal terms—e.g. by referring to unactualized states as ‘merely possible’ states—this is due to the basic law according to which the world can be in no more than one state at any instant of time.

Whether ray substantivalism is ultimately preferable to its competitors as an ontology of quantum theory is a question that can only be answered through a careful evaluation of the trade-offs between various metaphysical, epistemic, and pragmatic considerations. This question is unlikely to have easy answers. For example, it is not obvious whether ray substantivalism is ontologically simpler and more parsimonious than wavefunction monism.^46^ As we have seen, ray substantivalism involves some fairly significant ontological commitments: a fundamental space with the structure of Hilbert space as well as fusions of points in this space; State actuality; and the two fundamental almost-sameness relations on this space. But so does wavefunction monism. According to the only detailed intrinsic formulation of this view to date (Chen 2018), the fundamental posits include three-dimensional Euclidean space; *N*-membered collections of points in this space called *N*-*regions*, where *N* is the number of (non-fundamental) particles; as well as four primitive relations among *N*-regions: a dyadic relation ‘Amplitude-Geq’; a triadic relation of ‘Amplitude-Sum’; a triadic relation of ‘Phase-Clockwise-Betweenness’ and a tetradic relation of ‘Phase-Congruence’. While Chen’s proposal involves a greater number of primitive relations among (constructions out of) points of the fundamental space than does ray substantivalism, this is likely to be outweighed by the greater relational complexity required for an intrinsic account of Hilbert space—not developed in this paper—compared to axiomatizations of Euclidean space such as those by Tarski and Hilbert.^47^ These observations illustrate that comparing individual proposals even along a single dimension is not trivial. The question whether ray substantivalism achieves a better balance of the relevant metaphysical, epistemic and pragmatic considerations than its competitors strikes me as open.

## Conclusion

In this paper, I developed ray substantivalism, an ontology for non-relativistic quantum mechanics that vindicates symmetry fundamentalism. According to this ontology, state-space symmetries correspond to fundamental relations, whereas the entities we would ordinarily regard as the fundamental material building blocks of the world are ontologically derivative of these relations.

To some readers it might seem that we could have accomplished this much more quickly: simply by pointing to the relevant state-space automorphisms in the mathematical formalism and announcing that symmetry fundamentalism targets the physical entities (whatever they may be) to which these transformations correspond. This seems to be a common approach to ontological theorizing, and it essentially involves identifying the objects in the mathematical language of physics that are *physically* or *representationally* *significant*, in the sense of corresponding to some ontological item or other—by contrast to the ‘redundant’ mathematical entities, those that have no representational role to play.^48^ It is implicit in this approach that identifying which mathematical items are representationally significant is all that can be said by way of specifying an ontology: nothing further is to be gained by going ‘beyond’ the mathematical formulations in the way exhibited in this paper.

The approach I have taken is more ambitious: the relevant ontological primitives were specified *directly*, as it were, rather than by merely pointing to some mathematical items and saying that something or other corresponds to them.^49^ This approach doesn’t just highlight the distinctiness of the relevant physical items from their mathematical representations; it also vindicates the appropriateness of those representations for characterizing these items. This approach has clear advantages. First, we discovered that the physical structure of a non-relativistic quantum system without spin can be captured just in terms of two physical primitives: the almost-sameness relations that correspond to unitary spatial and momentum shift transformations. This is simpler than we might have expected: when it comes to position and momentum shifts, it seems natural to think that we would require one fundamental relation for each spatial direction and each shift amount, resulting in an enormous multiplication of ontological primitives. It seems likely that the significant gains in simplicity would have escaped us had we focussed just on determining which mathematical notions are representationally significant.

Second, when it comes to elucidating symmetry fundamentalism, the standard approach simply would not have provided us with anything close to the metaphysical insight we are looking for. There are settings in which we are sufficiently in control of the metaphysical background assumptions and the supporting intuitions to safely follow the representationalist strategy. I take it that most of us would have a fairly clear picture of what is being asserted by someone who tells us that the fundamental arena in which the history of the world unfolds is characterized by a three-dimensional manifold with a Euclidean metric. The claim that symmetries are fundamental and that every material object is ontologically derivative of them is quite different. Being told that a range of symmetry transformations are representationally significant and that they ‘correspond to’ physical items does not come close giving us to a clear picture of what the world would have to be like for that claim to be true. Indeed, the proponent of symmetry fundamentalism might run the risk of being profoundly misunderstood: it may seem like what’s being asserted is a form of pythagoreanism according to which the physical world is literally constituted by mathematical objects such as symmetry transformations. One way to avoid problems of this kind is to specify the relevant ontological primitives in the way I did in this paper.

This also illustrates the contrast between the results of this paper and an influential intellectual tradition known as ontic structural realism. At face value, ontic structuralism and the project of this paper have a lot in common. Roughly, ontic structuralists conceive of fundamental reality as populated by ‘structure’ rather than by individual objects such as particles, and symmetries are regarded as paradigm instances of this fundamental structure.^50^ However, ontic structuralist proposals suffer from the fact they are too often specified almost entirely at the level of the mathematical language given to us by physics. Indeed, while structuralists are quite liberal in their use of metaphysical notions such as necessitation,^51^ supervenience,^52^ determinables and determinates,^53^ constitution,^54^ ontological dependence,^55^ fundamentality,^56^ metaphysical grounding,^57^ and essence,^58^ these notions are ostensibly applied to straightforwardly mathematical objects. A few examples: French (2014, p. 283) proposes that “the Poincaré group is a determinable”. Roberts (2011, p. 50) argues for the claim that “the existing entities described by quantum theory are organized into a hierarchy, in which a particular symmetry group occupies the top, most fundamental position.” And McKenzie (2014a, pp. 21-2) entertains the hypothesis that “the Poincaré group [...] forms part of [an elementary particle’s] essence” which implies “the dependence of relativistic particles on the Poincaré group”.

This is not the place for a thorough assessment of these claims in particular or of the structuralist project in general. I merely want to register that those looking for an elucidation of a metaphysical vision along the lines of symmetry fundamentalism are unlikely to be satisfied with claims of this sort. Presumably, the subjects of the metaphysical attributes are not mathematical objects but the physical entities to which they correspond. But we are left in the dark as to what those physical entities are supposed to be. What we require isn’t more mathematical physics, but a transparent, precise and complete specification of the physical items to which the relevant mathematical items correspond. This is what I have endeavored to do in this paper.

Ray substantivalism is a novel ontology for non-relativistic quantum mechanics, one that is inspired by mathematical structures that have been and continue to be at the core of our best theories of fundamental physics. Although this is a significant result of its own, the explanatory power and theoretical utility of symmetry-fundamentalist ontological theorizing is likely to become fully transparent only once it is applied to the more sophisticated settings of quantum field theory and quantum gravity. For example: a symmetry-fundamentalist approach promises a metaphysical underpinning of the so-called ‘classification of elementary particle kinds’ in terms of the irreducible representations of Poincaré group as well as similar schemes involving the specification of color and flavor charge in terms of the group SU(3). It also provides a blueprint for the development of a fundamental ontology that details the physical counterparts of spin networks in loop quantum gravity.^59^ Although the scope of this paper was limited to non-relativistic quantum mechanics, its results are an important basis for future work on the symmetry-fundamentalist metaphysics of our best quantum theories.

## Footnotes

1 See (Martin 2003) for a survey of the rich history of symmetry techniques in 20th century.

2 (Weinberg 1987, p. 79).

3 (Weinberg 1987, p. 80).

4 (Weinberg 1992, pp. 138-9). Steven Weinberg has confirmed in personal correspondence that these remarks were intended as claims about fundamental physical reality, rather than merely about the epistemic or pragmatic significance of symmetry techniques.

5 (Heisenberg 1973, p. 273). Cf. (Heisenberg 1976, p. 924) and (Ne’eman and Sternberg 1991, p. 327)

6 In (Schroeren 2020), I develop a symmetry-fundamentalist ontology for classical particle mechanics.

7 The notion of a state-space symmetry is widely used by physicists, albeit occasionally under a different name: physicists sometimes refer to them as *Wigner symmetries* in recognition of Eugene Wigner (1931, 1968), the physicist who developed the first systematic account of this notion. Wigner symmetries are even mentioned in textbooks; for example, (Strocchi 2005; Moretti 2013). The notion of a state-space symmetry has also been deployed for philosophical purposes; for example, by John (2003, p. 341) and Simon (1996, p. 242).

8 For example: suppose that the dynamical evolution of two classical particles (labelled *A* and *B*) in one dimension is determined by the Hamiltonian $H= \frac{1}{2m}(p_A^2+p_B^2)$, where $p_A$ and $p_B$ denote the momentum coordinate functions of particles *A* and *B*, respectively. Shifts in the momentum of particle *A* are symplectomorphisms; and they are generated by the coordinate function $q_A$ representing *A*’s position coordinate. Moreover, shifts in the momentum of *A* are a dynamical symmetry only if $\{q_A,H\}=0$. But $\{q_A,H\}=\frac{p_A}{m}$, so momentum shifts of *A* are state-space symmetries but not dynamical symmetries.

9 For example, the fact that spin is discrete follows from the Peter-Weyl theorem on the assumption that rotations are state-space symmetries (i.e. unitary transformations); this is the only symmetry-related assumption required by the theorem (Folland 2015, p. 136).

10 This classification is due to Eugene Wigner (1939).

11 There is good evidence that this observation will also hold in the context of quantum gravity. In loop quantum gravity, for instance, the fundamental physical entities are mathematically characterized in terms of *spin networks*: graphs whose links are each assigned a Hilbert space on which the rotation group *SU*(2) acts unitarily. This state-space symmetry structure is then used to define the basic physical quantities *area* and *volume* (Rovelli 2011). As before, no assumption about dynamical symmetries is required for this definition.

12 For example, (Ballentine 2000, pp. 77).

13 A further step involves the specification of a differential equation that determines which curves through Hilbert space correspond to the dynamically allowed histories of the world: for example, the Schrödinger equation or a dynamical-collapse equation. Since the symmetries at issue in symmetry fundamentalism are state-space symmetries rather than dynamical symmetries, this aspect of the formalism is not directly relevant to the project in this paper.

14 (Schaffer 2009; deRosset 2010; Audi 2012; Dasgupta 2014).

15 Cf. (Ashtekar and Schilling 1999).

16 Strictly speaking, eigenvectors of unbounded Hermitian operators on ${\mathcal {H}}$ (such as those associated with the position and momentum degrees of freedom) are not elements of ${\mathcal {H}}$ but of the *rigged* Hilbert space associated with ${\mathcal {H}}$; i.e the triple consisting of ${\mathcal {H}}$, a dense subspace $\Phi \subset {\mathcal {H}}$, and its dual $\Phi ^*$ such that $\Phi \subset {\mathcal {H}}\subset \Phi ^*$. In this more rigorous setting, position and momentum eigenstates are ‘bras’, complex linear functionals that live in $\Phi ^*$ and that combine with ‘kets’ to yield a complex number. Here, I will follow the vast majority of discussions in the philosophy of quantum mechanics and ignore these technicalities. However, none of these technical complexities seem insurmountable: there is no obvious reason why it should not be possible in principle to specify ontological primitives that capture the structure of a rigged Hilbert space. This is a project I will not pursue here.

17 The relevant details can be found in (Schroeren 2020, pp. 12–21).

18 Since vector substantivalism quantifies over times, it presumes something like a B-theoretic account of time. An A-theoretic variant of vector substantivalism can be obtained by replacing quantification over instants of time with propositional tense operators such as ‘it will always be the case that...’, ‘it will at some time be the case that...’ etc, and by replacing the Point actuality relation with a monadic property ‘...is actual’.

19 Conceiving of Points as first-order individuals is not the only way to implement fundamentalism about the physical counterparts of Hilbert space vectors. The first alternative is to posit a fundamental first-order individual, ‘the world’, and to think of Points as *relations* such that necessarily, for every instant of time, there is exactly one such relation that obtains between the world and that instant. The A-theoretic version of this view regards Points as fundamental monadic properties such that, necessarily, the world always instantiates some Point (and no more than one Point). A second alternative is to regard Points as monadic properties of instants of time such that, necessarily, for every instant of time there is exactly one such property it instantiates. The A-theoretic version can be thought of as a kind of *propositionalism*: the view that Points are propositions or states of affairs such that, necessarily, there is always some such proposition (and no more than one such proposition) that is true or some such state of affairs that obtains. This choice point raises deep foundational issues that are beyond the scope of this paper; cf. (Bacon 2019).

20 See (Chen 2018) for recent work on nominalizing quantum theory.

21 Indeed, as noted by Scott (1959, p. 53), it is doubtful whether there is any intrinsic difference between distinct infinite-dimensional Hilbert spaces.

22 For systems with spin, the Hermitian operators corresponding to the spin degrees of freedom characterize the *kind* of property that the fundamental field assigns to every point: namely, properties mathematically specified in terms of spinors rather than in terms of complex numbers.

23 (Stone 1932).

24 The duality encoded in Stone’s theorem allows for a variant of vector substantivalism according to which the fundamental physical relations among Points correspond to Hermitian operators rather than to state-space symmetries. While potentially interesting, this version of vector substantivalism would not be an instance of symmetry fundamentalism.

25 The term ‘wavefunction realism’ was originally reserved for the configuration-space realist variant of the view. More recently, usage seems to have expanded to include any view according to which the wavefunction represents something “objective and mind-independent” (Chen 2019). Here, our discussion is limited to variants of wavefunction realism that posit a real physical field on some fundamental space.

26 The theories I call ‘local beable theories’ are distinct from theories proposed from as part of the so-called *primitive ontology* approach. According to the latter, the local beables are the *only* fundamental material constituents of the world, whereas the wavefunction is understood as part of the nomological structure of the world; cf. (Allori 2013).

27 (Albert 1996; Loewer 1996; Ney 2012; North 2013).

28 (Belot 2012; Chen 2017; Hubert and Romano 2018); cf. (Forrest 1988).

29 Note that there are versions of Bohmian mechanics that are not wavefunction realist. According to one proposal, the wavefunction has nomological status; on another, the wavefunction corresponds is a *sui generis* entity; (Maudlin 2013); cf. (Chen 2019).

30 Although the official ontology for EQM is spacetime-state realism (more on which below), EQM has often been introduced, developed, and criticized on the assumption of a configuration-space-realist wavefunction monist ontology; for example, (Brown and Wallace 2005; Saunders 2010; Maudlin 2010; Ney 2013). To my knowledge, a multi-field-realist wavefunction monist ontology for EQM has not been explicitly developed in the literature, though it is identified as a theoretical possibility in (Chen 2019, p. 10).

31 Consider a single-particle system in three-dimensional Euclidean space, characterized in terms of ${\mathbb {R}}^3$, and let $\hat{{\mathbf {x}}}$ be the position operator with eigenvalues in ${\mathbb {R}}^3$: if $\hat{{\mathbf {x}}}|\vec {x}\rangle = \vec {x}|\vec {x}\rangle$ then $\vec {x}\in {\mathbb {R}}^3$. The standard action of *SO*(3) on ${\mathbb {R}}^3$ is a map $\phi : SO(3) \times {\mathbb {R}}^3\rightarrow {\mathbb {R}}^3$, $(R, \vec {x})\mapsto \phi (R, \vec {x})= R \vec {x}$ where *R* are real invertible $3\times 3$ matrices such that $RR^T= \mathbb {1}$ and $detR= 1$. A unitary action $U : SO(3)\times {\mathcal {H}}\rightarrow {\mathcal {H}}$, $(R,|\psi \rangle )\mapsto U(R)|\psi \rangle$ on the one-particle Hilbert space ${\mathcal {H}}$ is then defined via $U(R)|\vec {x}\rangle = |R\vec {x}\rangle$.

32 (Wallace and Timpson 2010); cf. (Swanson 2018).

33 (Wallace 2012, p. 296).

34 For example, David Wallace explicitly rejects what he refers to as ‘Hilbert-space-vector realism’ (Wallace 2021, p. 69) and ‘Hilbert space realism’ (Wallace 2013, p. 216).

35 Cf. (Wallace and Timpson 2010; Swanson 2018).

36 Moreover, note that it would not help to put such entities in ‘by hand’, as it were. Wavefunction realist and spacetime-state realist theories are constructed to be ontologically complete in the sense that every relevant non-fundamental proposition is fixed by some proposition that is just about the fundamental physical field, and perhaps also about the local beables. The physical counterparts of symmetries would therefore be ontological ‘danglers’: propositions about them would not be required to fix the non-fundamental.

37 (Maudlin 2019).

38 (Albert 1996; Ney 2012; North 2013).

39 (Wallace 2008; Myrvold 2018).

40 There are at least two prominent wavefunction-monist approaches to the quantum measurement problem. First, notwithstanding his opposition to wavefunction realism (Wallace 2021), David Wallace’s many-worlds theory (Wallace 2012) can be straightforwardly understood in terms of a wavefunction-monist ontology. Second, the original version of GRW-dynamical collapse theory is wavefunction monist; (Ghirardi et al. 1986). (Ney and Albert 2013) contains further discussions.

41 (Wallace 2013, p. 216); (Wallace 2021, p. 70). But note some favorable mentions of such views in (Ladyman 2010, p. 157) and (French 2013, p. 86). Cf. (Bueno 2011, p. 93n13).

42 (Hawthorne 2010, p. 145).

43 (North 2013, p. 192).

44 ibid.

45 The parallels between possible worlds and instantaneous states are examined in (Williamson 2016).

46 I thank an anonymous reviewer for inviting me to elaborate on this issue.

47 (Hilbert 1899; Tarski 1959).

48 (Maudlin 2018). For a criticism of this approach from a different angle, see (Dewar 2017).

49 This approach is inspired by nominalistic constraints on ontological theorizing, according to which physical ontology must be specified without invoking mathematical entities at all; cf. (Field 1980; Arntzenius and Dorr 2012; Chen 2018). However, limitations of space prevent me from fully complying with nominalistic strictures in this paper. Instead, I have provided a nominalistic characterization of merely one part of the ontology: the part concerned with the fundamental items corresponding to mathematical symmetry transformations.

50 (Ladyman 1998; Castellani 1998b; Brading and Castellani 2003; Kantorovich 2003; Ladyman et al. 2007; Roberts 2011; French 2014; McKenzie 2014a; Brading et al. 2017).

51 (Berenstain and Ladyman 2012).

52 (Ladyman et al. 2007; Wolff 2011; Nounou 2012).

53 (French 2014, Section 10.7-8).

54 (Castellani 1998a).

55 (Wolff 2011; McKenzie 2014b).

56 (Ladyman 1998; Ladyman et al. 2007; Roberts 2011; McKenzie 2014b, a; French 2014).

57 (French 2014, pp. 169,283).

58 (McKenzie 2014b).

59 Cf. footnote 11.
